# Comparison of the Effectiveness of Single-Visit and Multi-Visit Root Canal Treatment on Healing Intraoral Sinus Tract

**DOI:** 10.1155/ijod/4200682

**Published:** 2025-07-17

**Authors:** Fatma Begüm Peker, Mehmet Burak Güneşer

**Affiliations:** Department of Endodontics, College of Dentistry, University of Bezmialem Vakif, Istanbul, Türkiye

**Keywords:** multi-visit endodontic therapy, postoperative pain, single-visit endodontic treatment, sinus tract management

## Abstract

**Objective:** This research sought to investigate and compare the levels of postoperative discomfort and the rate of intraoral sinus tract healing in teeth affected by chronic apical periodontitis, treated either through single-visit (SV) root canal therapy (RCT) or multi-visit (MV) therapy employing calcium hydroxide as an intracanal medicament.

**Materials and Methods:** A total of 60 participants, aged between 18 and 65 years, with single-rooted teeth exhibiting single canals, radiographic evidence of apical periodontitis, and associated intraoral sinus tracts, were included in the study. The participants were randomly divided into two treatment groups: Group 1 underwent SV therapy, while Group 2 received MV therapy. In the MV group, calcium hydroxide was introduced as an intracanal dressing following the preparation phase, and the root canal obturation was performed in a subsequent session after 1 week. In the SV group, obturation was completed in the same session. For the MV group, calcium hydroxide was removed during the second visit, the irrigation procedure was repeated, and obturation was finalized. Pain levels were measured postoperatively using a visual analog scale (VAS). Participants documented their pain at 6 and 12 h following treatment, as well as daily for the next 2 days. In the MV group, pain was additionally evaluated at the beginning of the second session, and patients were asked to report any pain experienced during the interval between visits. Follow-up appointments were conducted to assess the progress of sinus tract healing, which was defined as the complete closure or resolution of the tract over a 14-day period. Data were analyzed using the two-sample proportion test and Pearson's chi-square test, with statistical significance set at *α* = 0.05.

**Results:** The findings indicated no statistically significant differences between the two groups regarding the frequency of postoperative pain (*p* > 0.05). Median scores showed that VAS scores were highest after 6 h (Mdn = 3, IQR = 5.25), followed by 12 h (Mdn = 3, IQR = 6), after 24 h (Mdn = 0, IQR = 3.25), and after 48 h (Mdn = 0, IQR = 3). In both groups, pain levels at the 6^th^ h were found to be higher than at the 48^th^ h (*p* < 0.05). Mean recovery time was 6.8 days for the SV group and 6.56 days for the MV group. The duration required for sinus tract healing showed no significant variation between the SV and MV treatment modalities (*p* > 0.05).

**Conclusion:** Both SV and MV root canal treatments (RCTs) yielded comparable outcomes in terms of postoperative pain levels and the time necessary for sinus tract resolution, with no statistically significant differences observed between the two approaches. The observed similarity in healing parameters and pain levels suggests that both treatment methods demonstrate comparable clinical effectiveness.

## 1. Introduction

Apical periodontitis is a chronic inflammatory condition affecting the periradicular tissues, primarily triggered by microbial invasion of the pulp and subsequent necrosis [1, 2]. Among its various clinical manifestations, chronic apical abscesses are frequently encountered in endodontic practice. These lesions typically evolve through a prolonged inflammatory process and often present asymptomatically due to continuous drainage via sinus tracts [3]. The diagnostic challenge increases when sinus tracts manifest in atypical intraoral or extraoral locations, requiring both clinical experience and radiographic confirmation for accurate identification [4].

Histologically, chronic periapical lesions may develop into granulomas or cysts, indicating an ongoing balance between tissue destruction and repair [5]. Sinus tracts commonly appear on the buccal mucosa and are often associated with significant periapical radiolucency [6]. Studies have reported a variable incidence of sinus tracts in apical periodontitis, ranging from 8.5% to 18%, emphasizing the need for prompt recognition despite their often silent clinical presentation [7, 8].

While the presence of a sinus tract might suggest a more complex infection, several studies have shown that it does not necessarily compromise the prognosis of endodontic treatment if appropriate cleaning, shaping, and sealing are achieved [8–11]. However, the ideal treatment protocol—whether single-visit (SV) or multi-visit (MV)—remains under debate, particularly in teeth with sinus tract involvement. Historically, treatment over multiple visits using intracanal medicaments, such as calcium hydroxide was considered the standard approach. However, with advances in rotary instrumentation and irrigation, many clinicians now prefer completing root canal therapy (RCT) in a single session [12, 13].

Despite numerous investigations comparing SV and MV protocols, the literature offers inconsistent findings, especially concerning postoperative pain and sinus tract resolution. Some studies report no differences in pain or healing outcomes [12], whereas others advocate for MV treatment in cases of extensive infection. The variation in methodology, sample populations, and outcome measures across studies contributes to this ambiguity.

Therefore, this study aims to evaluate whether the number of treatment visits—single versus multiple—affects postoperative pain levels and sinus tract healing time in patients with chronic apical abscesses. We hypothesized that there would be no statistically significant difference between the two approaches regarding (1) postoperative pain levels over a 48-h period and (2) the time to complete sinus tract resolution.

## 2. Patients and Methods

This clinical study involved 60 patients requiring nonsurgical RCT. Ethical clearance was granted by the Ethics Committee of Bezmialem Vakif University (approval number: E.131495), and informed consent was obtained from all participants prior to enrollment. Eligible patients were between 18 and 65 years old and presented with teeth characterized by a single root and single canal, alongside radiographic signs of apical periodontitis and the presence of intraoral sinus tracts.

The selection of cases was based on specific inclusion criteria. Eligible teeth exhibited a sinus tract along with radiographic signs of periradicular bone loss. Only teeth diagnosed with pulp necrosis through vitality testing were considered. Additionally, the teeth had to be restorable and deemed suitable for endodontic treatment. Cases with a mobility score below three and periodontal probing depths not exceeding 4 mm were included.

Patients were excluded if they had significant systemic conditions, vertical root fractures, retreatment cases, calcified canals, internal or external root resorption, roots with open apices, or nonrestorable teeth. Additionally, patients experiencing preoperative pain or those who had taken preoperative medication (e.g., analgesics) within at least 12 h before treatment, which could influence pain perception, were not included.

Before proceeding, all participants received detailed information about the available treatment options, including potential benefits and risks, and provided informed consent for both the evaluation and treatment of their teeth. Participants were randomly allocated into two groups. Block randomization was used as the randomization method. Group 1 underwent SV RCT, while Group 2 received MV treatment.

### 2.1. Endodontic Treatment Protocol

For each patient, a gutta-percha cone was inserted into the sinus tract and traced radiographically to identify the involved tooth. This was followed by thermal pulp testing to assess the vitality of the implicated tooth and confirm its association with the sinus tract. Local anesthesia (Ultracaine DS Forte; Aventis Pharma, Istanbul, Turkey) was administered, and the operative field was isolated using a rubber dam to ensure sterility. Access cavities were created with sterile diamond burs. The working length of the canals was established using an electronic apex locator (Propex Pixi, Dentsply Sirona, Ballagues, Switzerland) and confirmed radiographically, with a #15 K-file.

Root canal preparation was performed using nickel-titanium rotary instruments (VDW GmbH, Munich, Germany) in accordance with the manufacturer's instructions. The canals were shaped with size #40 rotary files featuring a taper of 0.04. Root canals were irrigated with 2.0 mL of 2.5% NaOCl (Wizard, Ankara, Turkey) using a 30-gauge endodontic needle (Sybron Endo, Orange, CA, USA) after each instrument. The same treatment protocol was administered in both groups.

#### 2.1.1. SV Group

After completion of root canal instrumentation, the final irrigation protocol consisted of sequential rinsing with 5 mL of 2.5% sodium hypochlorite (Wizard, Ankara, Turkey), 5 mL of 17% EDTA (Werax, Izmir, Turkey), and 5 mL of saline. In the SV group, obturation was carried out in the same session immediately after final irrigation, using gutta-percha and an epoxy resin-based sealer (Ah Plus, Dentsply Sirona) with the cold lateral compaction method. Radiographs were obtained to verify the adequacy of canal obturation. The teeth were subsequently restored with composite resin material (Z250, 3M ESPE, USA), and occlusal adjustments were made as necessary to optimize functional outcomes.

#### 2.1.2. MV Group

For the MV group, calcium hydroxide paste (Kalsin, Spot Diş Deposu, Izmir, Türkiye) was introduced as an intracanal medicament using a lentulo spiral (size 40, Mani Inc., Tochigi, Japan) following canal shaping. The final treatment was completed during a second session scheduled 1 week later.

Obturation was performed during the second session after calcium hydroxide removal and a repeat of the final irrigation steps. The root canal fillings and coronal restorations were executed using identical methodologies and materials as described for the SV group.

### 2.2. Follow-Up Protocol

Postoperative pain was evaluated using a visual analog scale (VAS) at predetermined intervals (6, 12, 24, and 48 h) after treatment. Patients received detailed instructions on how to utilize the VAS forms and were asked to record their pain levels at 6, 12, 24, and 48 h postoperatively. In cases where the VAS forms were not returned, patients were contacted by telephone to obtain the missing data.

In the MV group, additional assessments of pain were conducted at the beginning of the second treatment session. Participants in this group were also asked to report any pain experienced during the interval between appointments.

The sinus patency was checked by two blinded evaluators. The provider of the treatment did not take part in the evaluations. Follow-up visits were arranged for all patients, and healing progress was monitored until the sinus tracts were confirmed to be fully resolved. Sinus healing was defined as the complete closure or disappearance of the sinus tract.

### 2.3. Sample Size and Statistical Analysis

The G^*⁣*^*∗*^^Power 3.1.9.4 program was used to determine the sample size. A minimum of 30 participants was estimated to be necessary, assuming a Type I error (*α*) of 0.05, a power (1-*β*) of 0.80, and an effect size of 0.53. The data were tabulated and described as percentages across different outcome categories. Treatment outcomes were categorized into two groups: successful (healed) and unsuccessful (diseased). For comparison of categorical variables, either Fisher's exact test or the chi-square test was used, depending on expected frequencies. The Shapiro–Wilk test was applied to assess the normality of continuous variables. As postoperative pain scores were not normally distributed, nonparametric tests (Mann–Whitney *U*) were employed. Postoperative pain assessments were conducted at multiple time points: 24 h, 48 h, and 7 days. To evaluate changes in pain levels within the same patients over time, the Wilcoxon signed-rank test was used for paired comparisons. For between-group comparisons at each time point, the Mann–Whitney*U* test was applied as an unpaired nonparametric test. All statistical tests were two-tailed with a significance threshold of *p* < 0.05. Pain scores were assessed at different time points between groups, and as no repeated measures within individuals were analyzed, unpaired tests were deemed appropriate. Potential outliers were evaluated using boxplots and *Z*-scores. Outliers were retained in the analysis as they were not deemed to be data entry errors and had clinical justification. Confidence intervals (95%) were reported for effect size estimates to provide insight into the precision and clinical relevance of findings. No regression analyses were performed; therefore, causal inferences were not drawn. All results are interpreted as associations rather than causal relationships.

## 3. Results

A total of 60 patients participated in this study, consisting of 37 women (61.6%) and 23 men (38.4%), with ages ranging from 18 to 65 years. Of the treated teeth, 39 (65.0%) were located in the maxilla, and 21 (35.0%) were in the mandible. The majority of sinus tracts were located on the buccal aspect, with maxillary lateral incisors being the most commonly affected teeth.

Postoperative pain levels following RCT were assessed at 6, 12, 24, and 48 h using VAS forms. The normality of VAS scores for both the SV and MV groups was tested using the Shapiro–Wilk test, which showed evidence of non-normality for all time periods (*p* < 0.001).

The distribution of pain incidence at 12 h was as follows: 36 patients (60%) experienced no pain, four patients (6.7%) reported mild pain, one patient (1.7%) reported moderate pain, and 19 patients (31.7%) reported severe pain.

### 3.1. Postoperative Pain

A Friedman test was performed to examine whether there was a difference in VAS scores across the four time periods (6, 12, 24, and 48 h). The results revealed a significant effect of time on VAS scores (*p* < 0.05). Postoperative pain levels at 6, 12, 24, and 48 h were generally comparable between the SV and multiple-visit groups, with no statistically significant differences observed, except for the interval between the 6^th^ and 48^th^ h (*p* < 0.05) (Table 1).

#### 3.1.1. SV Group

Median VAS scores were highest at 6 h (Mdn = 3, IQR = 5.25), followed by 12 h (Mdn = 3, IQR = 6), 24 h (Mdn = 0, IQR = 3.25), and 48 h (Mdn = 0, IQR = 3). Post hoc analysis using Wilcoxon signed-rank tests with a Bonferroni correction indicated that pain levels at 6 h (Mdn = 3) were significantly higher than at 48 h (Mdn = 0, p < 0.05, *r* = 0.45) (Table 1).

#### 3.1.2. MV Group

Median VAS scores were highest at 6 h (Mdn = 2.5, IQR = 6.25), followed by 12 h (Mdn = 0, IQR = 5), 24 h (Mdn = 0, IQR = 4.25), and 48 h (Mdn = 0, IQR = 0). Post hoc analysis with a Bonferroni correction revealed that pain levels at 6 h (Mdn = 2.5) were significantly higher than at 48 h (Mdn = 0, *p* < 0.05, *r* = 0.41) (Table 1).

### 3.2. Recovery Time of Sinus Tract

Sinus tracts resolved within 3–14 days in all patients, except for one case in the MV group (a maxillary canine), in which the sinus tract remained open for more than 2 weeks. To assess differences in healing time, the Shapiro–Wilk test indicated non-normality in both groups (*p* < 0.001), justifying the use of the Mann–Whitney *U* test. As shown is Table 2, the sinus tract healing times did not differ significantly between the SV group (M ± SD: 6.80 ± 4.08 days) and the MV group (M ± SD: 6.56 ± 3.85 days), as confirmed by the Mann–Whitney *U* test (*U* = 441, *p*=0.886). The median healing time for both groups was 7 days (Table 2).

## 4. Discussion

One of the most common challenges faced by clinicians during RCT is managing chronic apical abscesses, which are a well-recognized sign of progressive infection, often presenting with an intraoral or extraoral sinus tract. When infection escapes through the root apex, it follows the path of least resistance through bone and soft tissue. Once the cortical plate is breached, the exit point of the sinus tract is determined by several factors, including the placement of muscle attachments, fascial sheaths, the position of the tooth in the dental arch, bone thickness, and the distance to intraoral or extraoral environments [14]. Additionally, factors such as gravity and the virulence of the microorganisms, can further influence the exit route.

In cases involving preoperative apical pathosis, MV endodontic treatment is often preferred due to its stronger biological basis and greater potential for microbial control. This approach facilitates more thorough disinfection, with calcium hydroxide used as an intracanal medicament between visits to neutralize toxins and bacteria, which may improve healing outcomes [15]. The multistep treatment process allows for better management of infection and inflammation, especially in more complex cases. However, the debate over the merits and drawbacks of SV versus MV treatments persists, with both approaches offering distinct advantages and challenges. While MV treatment is traditionally favored for more complicated cases, its effectiveness is still evaluated in comparison to the SV treatment [5, 16, 17].

This study investigated postoperative pain and healing outcomes in SV versus MV endodontic treatments for teeth with chronic apical abscesses associated with sinus tracts. Remarkably, all intraoral sinus tracts resolved within 14 days, except for one case involving a maxillary canine treated with the MV approach, in which the sinus opening remained open beyond this period. Retrospective studies have demonstrated that sinus tracts resolving within 1 month are linked to superior long-term bone healing and an overall improved prognosis [18], highlighting the clinical relevance of early resolution. Similarly, a case study reported initial signs of bone healing within 2 weeks, further emphasizing the importance of timely intervention [19].

These results align with prior findings, yet the persistence of the sinus tract in the case of the maxillary canine, despite MV treatment, suggests that certain factors—such as the specific anatomy of the tooth, infection severity, or patient-specific variables—may influence treatment outcomes. Further exploration of these variables may help explain discrepancies between individual cases and the general trends observed in this study.

To further evaluate therapeutic efficacy, cases exhibiting persistent clinical or radiographic signs of pathology beyond the 14-day observation period were monitored for an additional month. The lack of significant changes during this extended follow-up reinforced the 14-day mark as a critical threshold for assessing treatment success and determining the necessity for further clinical intervention. These findings highlight the pivotal role of early sinus tract closure in enhancing endodontic outcomes and long-term prognosis.

When sinus tracts originate from pulpal pathology, they typically demonstrate a favorable response to adequate nonsurgical endodontic intervention. Evidence suggests that conservative RCT alone is often sufficient to achieve complete resolution, eliminating the need for surgical intervention and supporting a positive long-term prognosis [20–23]. This further underscores the potential benefits of optimizing nonsurgical treatment protocols, especially for managing cases with chronic apical abscesses and sinus tracts.

Postoperative pain remains a frequent concern for patients undergoing RCT. Studies have demonstrated that both SV and MV RCTs can result in postoperative pain, although its incidence and severity vary widely. Factors influencing postoperative pain include the severity of infection, case complexity, the patient's pain tolerance, and the clinician's technique [24–28]. A systematic review reported postoperative pain prevalence ranging from 3% to 58% [29]. In our study, postoperative pain was observed in 52.77% of patients, which aligns with the reported range in the literature. This broader understanding of postoperative pain lays the foundation for evaluating differences between SV and MV approaches.

Furthermore, prior studies comparing SV and MV treatments found no significant differences in postoperative pain levels [12]. Similarly, our findings showed no statistically significant differences in postoperative pain between SV and MV treatment groups. A review of randomized controlled trials has not demonstrated the superiority of single-session RCT over multi-session treatment. However, SV RCT has been associated with increased postoperative pain 1 week after treatment compared to RCT completed over multiple visits [30].

The consistency of our results with previous research supports the notion that both SV and MV approaches are viable options for managing chronic apical abscesses with sinus tracts, allowing clinicians to tailor treatment plans based on patient-specific factors and clinical circumstances. For example, SV treatments might be more suitable for patients seeking immediate relief, whereas MV treatments may provide greater flexibility for complex cases requiring multiple interventions.

However, the interpretation and generalizability of these findings must be considered in light of several limitations. Our relatively small sample size—primarily due to specific inclusion criteria focusing on single-rooted teeth undergoing initial RCT with sinus patency—limits the ability to extrapolate results to broader populations, such as patients with multirooted teeth or retreatment cases. Additionally, the observational study design precluded control over variables like practitioner skill, patient compliance, and comorbidities, all of which may influence treatment outcomes. The follow-up period of 1 month further restricts assessment of long-term outcomes, such as retreatment needs or posttreatment complications.

To address these constraints and strengthen evidence, future large-scale, multicenter studies with extended follow-up periods are warranted. Such research would enhance generalizability and provide deeper insight into sustained treatment success for chronic apical abscesses.

## 5. Conclusion

In conclusion, this study found no significant differences between SV and MV RCT regarding sinus tract healing time or postoperative pain levels. Both approaches were effective in managing chronic apical abscesses associated with sinus tracts. The findings further substantiate that the presence of a sinus tract does not appear to adversely affect the prognosis of RCT.

Although based on a limited sample size, the findings suggest that neither single-session nor multi-session endodontic treatment provides distinct advantages in terms of short-term clinical outcomes, patient comfort, or postoperative pain management. This underscores the importance of tailoring treatment plans to individual patient factors, while highlighting the need for further research to establish evidence-based protocols for managing chronic apical abscesses with sinus tracts.

Practical Implications: The findings from this study have practical implications for clinicians, as they suggest that both treatment approaches—SV and MV—can effectively manage chronic apical abscesses with sinus tracts. The absence of significant differences in healing time or postoperative pain levels means that clinicians can choose the approach based on the individual needs of the patient, their clinical judgment, and other practical considerations, such as patient comfort, treatment complexity, and time constraints.

Significance in Broader Research Context: This study contributes to the ongoing discussion regarding the optimal management of chronic apical abscesses and sinus tracts. It reinforces the notion that sinus tract resolution within a short period is critical for improving long-term prognosis and highlights the effectiveness of nonsurgical endodontic treatments. The findings also align with existing literature suggesting that there is no clear superiority of one treatment approach over the other in terms of postoperative pain, but underscore the importance of individualized treatment planning based on patient and case-specific factors.

## Figures and Tables

**Table 1:** Comparison of postoperative pain levels between single-visit and multiple-visit root canal treatments at 6, 12, 24, and 48 h postoperatively.

	Group	M ± SD	Mdn	IQR (Q1:Q3)	Min:Max	*p*-Value
6 h	Single visit	2.97 ± 2.60	3	5.25 (0:5.25)	0:8	0.926
	Multiple visits	2.90 ± 3.05	2,5	6 (0:6)	0:8	—

12 h	Single visit	2.80 ± 2.76	3	6 (0:6)	0:7	0.484
	Multiple visits	2.37 ± 2.71	0	5 (0:5)	0:7	—

24 h	Single visit	1.70 ± 2.13	3	3.25 (0:3.25)	0:6	0.625
	Multiple visits	1.57 ± 2.71	0	4.25 (0:4.25)	0:8	—

48 h	Single visit	1.07 ± 1.85	0	3 (0:3)	0:5	0.497
	Multiple visits	0.73 ± 1.55	0	0 (0:0)	0:5	—

*Note:* A statistically significant difference in postoperative pain levels was observed between the 6^th^ and 48^th^ h, while no significant differences were found between the other time points.

**Table 2:** Comparison of sinus tract healing times between single-visit and multiple-visit root canal treatment.

Group	M ± SD	Mdn	IQR (Q1:Q3)	Min:Max	*p*-Value
Single visit	6.80 ± 4.08	7	4 (3:7)	3:14	—
Multiple visits	6.56 ± 3.85	7	4 (3:7)	3:14	0.886

*Note:* Healing outcomes between the two treatment groups were not significantly different (*p*=0.886).

## Data Availability

The data that support the findings of this study are available upon request from the corresponding author. The data are not publicly available due to privacy or ethical restrictions.
